# Processing Factors Affecting the Phytochemical and Nutritional Properties of Pomegranate (*Punica granatum* L.) Peel Waste: A Review

**DOI:** 10.3390/molecules25204690

**Published:** 2020-10-14

**Authors:** Tandokazi Pamela Magangana, Nokwanda Pearl Makunga, Olaniyi Amos Fawole, Umezuruike Linus Opara

**Affiliations:** 1 Department of Botany and Zoology, Stellenbosch University, Private Bag X1, Matieland, Stellenbosch 7602, South Africa; tkmagangana@sun.ac.za (T.P.M.); makunga@sun.ac.za (N.P.M.); 2 Postharvest Technology Research Laboratory, South African Research Chair in Postharvest Technology, Department of Horticultural Sciences, Faculty of AgriSciences, Stellenbosch University, Private Bag X1, Stellenbosch 7602, South Africa; 3 Postharvest Research Laboratory, Department of Botany and Plant Biotechnology, University of Johannesburg, P.O. Box 524, Auckland Park, Johannesburg 2006, South Africa; olaniyif@uj.ac.za

**Keywords:** agriculture waste, antioxidant activity, horticultural processing, pomegranate peel, postharvest physiology, total phenolic content, value addition

## Abstract

Pomegranate peel has substantial amounts of phenolic compounds, such as hydrolysable tannins (punicalin, punicalagin, ellagic acid, and gallic acid), flavonoids (anthocyanins and catechins), and nutrients, which are responsible for its biological activity. However, during processing, the level of peel compounds can be significantly altered depending on the peel processing technique used, for example, ranging from 38.6 to 50.3 mg/g for punicalagins. This review focuses on the influence of postharvest processing factors on the pharmacological, phytochemical, and nutritional properties of pomegranate (*Punica granatum* L.) peel. Various peel drying strategies (sun drying, microwave drying, vacuum drying, and oven drying) and different extraction protocols (solvent, super-critical fluid, ultrasound-assisted, microwave-assisted, and pressurized liquid extractions) that are used to recover phytochemical compounds of the pomegranate peel are described. A total phenolic content of 40.8 mg gallic acid equivalent (GAE)/g DM was recorded when sun drying was used, but the recovery of the total phenolic content was higher at 264.3 mg TAE/g when pressurised liquid extraction was performed. However, pressurised liquid extraction is costly due to the high initial investment costs and the limited possibility of carrying out selective extractions of organic compounds from complex peel samples. The effects of these methods on the phytochemical profiles of pomegranate peel extracts are also influenced by the cultivar and conditions used, making it difficult to determine best practice. For example, oven drying at 60 °C resulted in higher levels of punicalin of 888.04 mg CE/kg DM compared to those obtained 40 °C of 768.11 mg CE/kg DM for the Wonderful cultivar. Processes that are easy to set up, cost-effective, and do not compromise the quality and safety aspects of the peel are, thus, more desirable. From the literature survey, we identified a lack of studies testing pretreatment protocols that may result in a lower loss of the valuable biological compounds of pomegranate peels to allow for full exploitation of their health-promoting properties in potentially new value-added products.

## 1. Introduction

The pomegranate (*Punica granatum* L.) (Lythraceae; formerly belonging to the Punicaceae family) is a fruit-bearing deciduous shrub or tree [1]. Native to Iran and Afghanistan, it is found growing wild in the outer hills and warm valleys of the Himalayas, and is often cultivated throughout northern India, but now pomegranate is grown commercially in various parts of the world, including South Africa, North Africa, South America, North America, the Middle East, Israel, and Australia [2,3,4].

**Figure 1:**
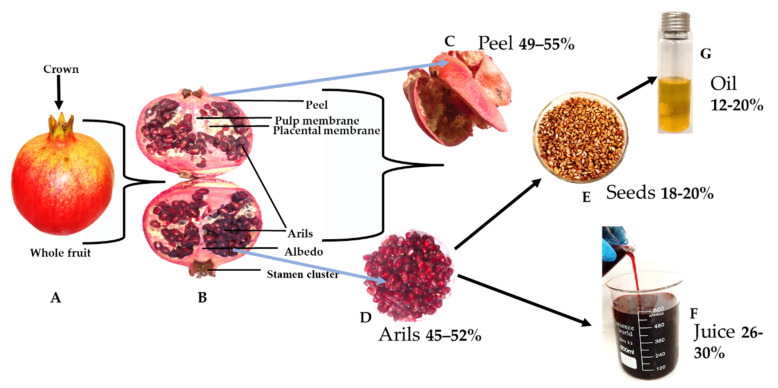
Botanical description of *Punica granatum* L. fruit: (**A**) whole fruit; (**B**) anatomical description of pomegranate fruit; (**C**) peel; (**D**) arils; (**E**) seeds; (**F**) juice; (**G**) oil.

The popularity of and demand for pomegranate are on the rise all over the world due to its multifunctionality and health-promoting effects in the human diet. The arils account for about 45–52% of the weight of the whole fruit [5], and have long been valued for their flavourful juice (Figure 1F). The peel accounts for about 49 to 55% of the weight of the fruit, depending on the cultivar [5]. The interior of the pomegranate fruit (Figure 1B) is separated by membranous walls (septums) and white spongy tissue chambers (locules) packed with arils containing soft or hard seeds and edible juice, which vary in colour from white to red, depending on the cultivar [6]. Pomegranate fruit has numerous seeds, which account for about 18–20% of the fruit weight and contain oil (Figure 1G). Pomegranate seed oil (PSO) accounts for about 12–20% of the fruit weight and also has many therapeutic properties [5].

The pomegranate peel, commonly considered as an agro-industrial waste, is a potential source of valuable secondary plant metabolites and nutrients. It has stronger biological activity than the pulp [7,8,9], yet often it is used as animal feed, whereby most is discarded, creating an environmental problem due to the moisture content it may carry. The strong biological activity is linked to its specific chemical composition, especially the secondary metabolites, such as flavonoids and plant phenolics [5,10]. Pomegranate peel could prove to be more beneficial in the form of food additives or supplements. By optimising food waste recovery strategies that could be used in the food and bioprocess industries, it also has the potential to produce innovative products that can meet the needs of the growing health market. For instance, in the area of nanotechnology, biosynthesis of biological nanoparticles using biological agents such as plant-based extracts has gained attention in the past decade [11]. Bio-based protocols for the synthesis of nanoparticles that are economical, environmentally friendly, and suitable for large-scale production under non-aseptic environments are in demand [12,13]. Pomegranate peel extracts are suggested to be good reducing agents that are used in the green synthesis of silver nanoparticles, as this technology has potential for creating materials, devices, and systems with fundamentally new functions and properties [13,14,15]. Silver nanoparticles may be utilised for a wide range of applications, including in electrochemistry, catalysis, bioengineering, cosmetics and food packaging, and environmental uses, and as antiseptic agents in the biomedical industry [13,14,16]. Goudarzi and Salavati-Niasari [17] investigated the use of pomegranate peel powders as new capping agents in the synthesis of CuO/ZnO/Al_2_O_3_ nanostructures to enhance the visible light photocatalytic activity. The authors concluded that in the presence of the pomegranate peel powders via the facial and green methods, CuO/ZnO/Al_2_O_3_ nanoclusters were successfully synthesised with photocatalytic potential. This is useful in the removal of azo dye contaminants, such as methyl orange, methylene blue, and methyl red, which are harmful to the environment and not biodegradable. The use of pomegranate peel powder in the synthesis of CuO/ZnO/Al_2_O_3_ nanoclusters shows potential for the reuse of coloured wastewater (azo dyes) in the textile industry.

Due to the multifunctionality and nutritional properties of pomegranate fruit peel, numerous industries are interested in its special benefits and new ways of using the fruit peel. In the food and beverage industry, pomegranate peel has been used for the fortification and formulation of food products such as cereal bars, beverages, ice cream, and yoghurts to enhance their functional properties [5,8,10,18]. The peel has been incorporated as an ingredient in many functional foods, dietary supplements, colourants, flavouring agents, edible coatings, and in food packaging films [19,20,21,22]. Nur Hanani et al. [23] aimed to develop an active packaging system based on gelatin–polypropylene bilayer films with different fruit peel powders of pomegranate, papaya, and jackfruit. All of those films containing fruit peel powders displayed antimicrobial and antioxidant activities, with pomegranate peel powder having the most notable effect. Similar findings in the food packaging industry pertaining to the valorisation of the pomegranate peel powder and its extracts have been reported by numerous other authors [24,25,26,27,28].

Pomegranate fruit peel extracts have been used as natural additives to increase the shelf life of food, including meat products. Kanatt et al. [29] assessed the antioxidant activity of pomegranate peel extract in two popular Indian chicken meat products. The addition of the pomegranate peel extract to the meat products enhanced their shelf life by 2–3 weeks during chilled storage. The shelf life of meat containing pomegranate peel extracts has been studied by several authors [22,30,31,32,33]. The fruit peel has high antioxidant and antimicrobial activities and may be used as an excellent natural additive for food preservation and for quality enhancement. The health-promoting benefits of pomegranate peel have prompted the food industry to focus on pomegranate-peel-containing food preparations, which include nutraceuticals, phenolic-enriched diets, and food supplements [8,29]. The nutritional profile of the pomegranate peel shows high levels of fibre, organic acids, polyphenols, minerals, vitamins, and proteins, as well as low calories, making the fruit peel attractive and profitable [8,10,34].

Apart from the rich benefits of fruit peel in the food industry, it also shows potential in the bioenergy sector as a cost-effective and environmentally friendly fuel [35,36,37]. In the dye industry, it has been used for the commercial production of ink and dye [38,39,40]. Furthermore, the pharmaceutical and cosmeceutical industries have used pomegranate fruit peel in creating therapeutic formulations and skin improvement creams [5,8]. The pomegranate peel is an inexhaustible resource with many potential functions in numerous industries; further exploration of the fruit peel may lead to new and novel ways of creating wealth from waste.

Globally, the pomegranate cultivation area is estimated to be around 300,000 hectares with a yield of 3 million metric tons [41], with India being the most important producer at 0.81 metric tons. The southern hemisphere contributes about 10% of the pomegranate fruit, and in 2018, South Africa produced 5547.1 tons of pomegranates [42]. There has been great commercial interest in the waste products from these super foods due to the high nutritional value they possess, making them economically attractive. Fruit and vegetable waste has been employed in various countries all over the world, including in numerous pomegranate-based foods and pharmacological and cosmetic products. Value addition can create different layers of industry and more opportunities, especially in developing economies. Additionally, the waste management of pomegranate peel could lead to major reductions in environmental burdens. To achieve this, consideration of the peel characteristics is needed to preserve the phytochemical and biological properties. Various technologies may be able to conserve the bioactive compounds of the pomegranate peel during processing. Drying and extracting plant material under different conditions can cause significant changes to the chemical profile and biological attributes of the secondary metabolites found in the peel. These metabolites are beneficial to the food, nutraceutical, and pharmaceutical industries. There is also a need to optimise fruit processing factors to obtain the maximum yield for value addition. Complete utilisation of resources reduces food waste and has positive impacts on food security, the economy, and the climate. Therefore, this review aims to discuss the processing factors (such as drying, extraction, and pretreatment) influencing the pharmacological, phytochemical, and nutritional properties of pomegranate peel. A general overview of the biological profile of pomegranate peel is provided, followed by an extensive synopsis of the diverse drying practices and extraction protocols that have been tested using pomegranate fruit peels.

## 2. Pharmacological, Phytochemical, and Nutritional Composition of Pomegranate Peel—An Overview

Pomegranates have been integral to different societies due to their medicinal value, and there is a rich cultural history of their relevance to society. There is growing evidence [8,9,43,44] linked to ethnopharmacological research on pomegranates. The ethnopharmacological profiles of the entire plant and its different parts (flowers, leaf, bark, juice, seed, and roots) are the reasons for its demand and renowned popularity over the ages, besides its role in art and religion. In Ayurvedic medicine, the pomegranate is considered “a pharmacy unto itself” [45]. The roots and bark exhibit healing properties against vermifuge and anthelmintic effects [46]. The flower buds are used to alleviate the symptoms of diarrhea, dysentery, and bronchitis; to reduce inflammation of the gums; and for the treatment of eyes, throats, and ulcers (Table 1).

Over the past few decades, scientific investigations have been undertaken to give a credible basis for the traditional ethnopharmacological uses of pomegranate (Table 1; [46,47,48,49,50,51,52,53,54]. The fruit is the most valuable part of the pomegranate tree, however, different parts contain valuable compounds, such as the peel, arils, and seeds. For example, Fawole et al. [8] outlined the antibacterial activity of peel compounds against selected microorganisms and showed that the vitamin C content could vary between the fruit peel and arils [9], highlighting the importance of examining different parts of the fruit in the search for bioactive compounds. The fruit is, thus, a rich source of bioactive compounds, such as polyphenols, which have antioxidant properties that are suggested to reduce the risk of disease in humans [45,55]. For years, the entire pomegranate fruit tree and its fruit extracts have been studied for their antidiabetic potential [45], anticancer properties [56,57], apoptotic and antigenotoxic potential [58], cardioprotective properties [57], antiobesity properties [59], and free radical scavenging properties [8,10] (Table 1).

Numerous other studies performed over the years have suggested that pomegranate extracts contain various pharmacological properties (Table 2; [8,9,43,44,60,61,62]). Although the edible part of the fruit—the arils in particular—are valuable and nutrient- and phenolic-rich, the non-edible peel fractions contain even higher amounts of valuable nutrients and stronger biologically active constituents when compared to the edible arils, in spite of these parts being considered as waste [7,8,34]. However, in most cases, the peel (outer thick skin or rind), which constitutes about 50% of the fruit weight, is discarded after juice processing [7,8,9].

### 2.1. Phytochemistry and Nutritional Constituents of the Pomegranate Peel

Pomegranate peel accounts for about 49–55% of the fruit weight, depending on the cultivar [5,34,63]. It contains numerous vitamins, minerals, and organic acids, dietary fibre, and phenolic compounds such as flavonoids, which include anthocyanins and flavonols; as well as condensed tannins such as proanthocyanidins and hydrolysable tannins, which include ellagitannins and gallotannins [5,8,34,55,64]. As a collective, these compounds exhibit strong antioxidant, antimicrobial, antimutagenic, cardio-protective, apoptotic, and antigenotoxic potential, which may produce ameliorating effects against numerous critical diseases [5,8,34,55,57,58,64]. Different parts of the pomegranate tree contain substantial amounts of the secondary compounds (Table 3) that are responsible for the reported biological activities (Table 2). The chemistry of pomegranate is complex and about 124 different phytochemicals can be found in the pomegranate fruit. From these, approximately 48 phenolic compounds, especially anthocyanins, hydrolysable tannins, hydroxycinnamic acids, gallotannins, and hydroxybenzoic acids, have been detected in the peel and other parts of the fruit [19,63,64]. Interestingly, the above phenolic compounds, particularly the hydrolysable tannins, are predominantly higher in the pomegranate peel as compared to other parts of the pomegranate fruit [5,8,19]. As a result of this, the phytochemical concentration of the peel is said to be effective without the addition of other fruit fractions [5,63]. Approximately 30% of all fruit contents of anthocyanidins are concentrated in the fruit peel [19]. All of these secondary compounds have a great role in the performance and potency of the antioxidant effect, flavour, texture, and colour of the whole fruit, which aid in its attractiveness in the market and exploitation of its biological activity [5,8,10]. The concentrations of these compounds and their strong biological properties depend on the cultivar, developmental stage of the fruit, environmental conditions, and processing method used to recover the phenolic compounds [3,4,19,34].

### 2.2. Organic Acids and Total Sugars

Organic acids (such as citric acid, acetic acid, gluconic acid, lactic acid, itaconic acid, and ascorbic acid), as well as total sugars, are in great demand worldwide, and as a result, are produced at high volume (millions of tons per year) [66]. They are used in the food and beverage industries as flavourings in various products [66,67,68] and have applications as preservatives in the medical industry, as organic acids inhibit microorganisms, prolonging the shelf life of products [66]. It is predicted that by the year 2030, almost 35% of chemical products will be generated in a biotechnological manner [66]. Numerous studies have been conducted on the biological roles played by citric acid, succinic acid, and various other organic acids obtained from several fruit peel sources [69,70,71,72,73,74]. This shows that there is an economical alternative and an environmentally friendly way to solve the problem of waste management of fruit peel.

Pomegranate peel is a good source of organic acids and many other nutritional components, which contribute to its popularity and demand in the market [3,4,6]. There are numerous organic acids that are present in the fruit peel, such as citric, malic, acetic, oxalic, tartaric, lactic, ascorbic, and fumaric acids, which have the potential for value addition in the market and are currently being studied by various authors for their biological activity, along with the ways to optimise their extraction during processing. For example, Dafny-Yalin et al. [67] studied the levels of organic acids in 29 pomegranate accessions and reported that citric acid was the major organic acid found in the peels of all accessions. Several other organic acids were detected in peel homogenates, including malic, succinic, and oxalic acids, while glucose and fructose were the main sugars present. These sugars varied significantly amongst the studied pomegranate (PG) accessions, ranging from 0.9 ± 0.05 g/100 g in accession PG 200–211 to 4.8 ± 0.28 in accession PG 203–214 for glucose, and from 0.9 ± 0.07 g in accession PG 200–211 to 6.6 ± 0.65 in accession PG 11415 for fructose. In addition, maltose was also detected at a range of 0.8 ± 0.04 mg/100 g in accession PG 206–217 to 48.9 ± 4.6 mg/100 g in accession PG 201–212, whereas sucrose was detected in very small quantities. Pande and Akoh [68] investigated six pomegranate cultivars, namely R19, R26, Cvg-Eve, North, Crab, and Cranberry, for the accumulation of organic acids in different parts of the fruit. Higher organic acids were evident in the peel compared to the leaf and seeds. Citric acid followed by malic acid were found to be the predominant organic acids in the peel, ranging from 507.0 ± 3.5 mg/100 g of the fresh weight (FW) in the North cultivar to 1678.2 ± 8.4 mg/100 g in the R26 cultivar, and from 93.0 ± 2.6 mg/100 g of FW in the North cultivar to 116.5 ± 2.4 mg/100 g of FW in R26 cultivar, respectively.

Numerous other works have noted the variations in phenolic compositions, organic acids, and sugars of the pomegranate fruit peel [64,75,76]. Variations of organic acids and total sugars may be due to several reasons, such as agro-climatic conditions, cultivar differences, fertilizers, irrigation, fruit maturity, storage, and postharvest treatments, all of which affect the quality attributes of the fruit. Pomegranate peel is a so-called “superfruit” waste product, representing a rich source of organic acids and total sugars, which are in great demand in the health and food industries worldwide. Biological production of these valuable substances from pomegranate peel waste provides opportunities to produce high quality, non-toxic, and moderately priced products that are beneficial to both the consumer and the industry. Whilst there are numerous products on the market produced from pomegranate peel, processing methods for the peel to obtain high-quality, phytochemically rich, and safe pomegranate products remains the number one aim for the industry.

### 2.3. Vitamin and Mineral Contents

Fruit peels are rich sources of vitamins and minerals and can be utilised as food, feed, and in many dietary ingredients after appropriate processing. Numerous studies conducted on fruit peel waste have shown that these waste products possess many biological activities, such as antioxidant, antimicrobial, and anticancer activities [7,8,9,72]. Vitamins and minerals are a class of essential nutrients in the body that are required for human health. Their consumption may prevent several deficiencies that can lead to various health disorders [77,78]. Minerals are divided into two groups—macronutrients and micronutrients. The former are required in greater amounts of approximately 100 mg per day and constitute 1% of body weight, while the latter are required in small amounts in the body and make up 0.01% of body weight [77]. After appropriate processing, peel waste can yield high amounts of vitamins and minerals, which are essential in human bodies, and also may be utilised in the nutraceutical, pharmaceutical, cosmeceutical, and food industries.

Pomegranate peel may be used as an ingredient in all of the above-mentioned industries, as it contains high amounts of essential nutrients [7,63,64,77]. Fawole and Opara [77] studied the compositions of trace and major minerals in the peels of seven pomegranate cultivars (namely Arakta, Bhagwa, Ganesh, Herskawitz, Ruby, Molla de Elche, and Wonderful) and highlighted significant differences amongst the profiled peels of the different cultivars in relation to their mineral content. In summary, the Bhagwa cultivar had the highest nitrogen, phosphorus, potassium, sulphur, and chlorine contents at 351.50, 33.03, 554, 19.43, and 122.96 mg/kg, respectively. The other cultivars, namely Ruby, Arakta and Wonderful, contained the highest calcium (60.65 mg/kg), magnesium (26.4 mg/kg) and sodium (117.40 mg/kg) levels, respectively. Kushwaha et al. [79] studied the nutritional composition of detanninated and fresh pomegranate (cv. Bhagwa) peel powders and indicated that the fresh peel powder had higher vitamin A at 14.06 µg/gm. This powder also contained minerals such as copper, iron, magnesium, potassium, and sodium at 6.2 µg/gm, 22.6 µg/gm, 1644.47 mg/kg, 16,237.4 mg/kg, and 763.66 mg/kg, respectively.

Ismail et al. [80] explored the nutritional potential of pomegranate peel (cv. Alipuri) as a source of antioxidants, dietary fiber, and minerals for use as a supplement for wheat flour to produce organoleptically acceptable and nutritionally enriched cookies. They recorded high calcium and potassium contents in the peel at 1192 and 2749.46 mg/kg, respectively. The addition of the pomegranate peel powder to the wheat flour resulted in significant improvements in the calcium and potassium contents of the pomegranate-peel-supplemented cookies of 46.90–175.41 and 327.26–598.61 mg/kg, respectively, at a replacement level of 7.5%. Numerous studies on pomegranate peel have shown the great potential for pomegranate peel extracts to be utilised for value addition as fortification agents in several industries due to their nutrient richness [5,7,19,63,64]. Dietary incorporation of the vitamins and minerals in pomegranate peel for value addition may prove to be a profitable way to use the peel waste.

## 3. Effects of Drying Methods

Pomegranate peel is high in moisture and cannot be stored for further utilisation without the application of a preservation method. This affects the quality of polyphenols at the extraction stage. Therefore, it is necessary to choose the right technique for processing that will not compromise the integrity of the product. The drying process is a dehydration technique and is a vital step in reducing the moisture content for preservation purposes, thus increasing the shelf life and directly influencing the physical and chemical changes of the products [81,82,83,84]. This is the most widely used method for the preservation of decomposable materials such as fruits and vegetables (including their waste products), which have huge commercial and industrial relevance [81,83].

Despite these benefits, the drying process may change the physical, nutritional, chemical, and biological quality parameters [9,34,82]. Numerous conventional methods of drying have been assessed in the literature, such as sun drying, solar drying, freeze-drying, microwave drying, and fluidised bed drying (Table 4; [9,18,34,85,86,87,88,89]). It is important to select appropriate drying techniques in order to form a good product. The selection of the proper drying method may be influenced by several factors, such as the product type, availability of the drying equipment, drying conditions, cost of the drying operation, and the drying efficiency [81,84].

### 3.1. Sun Drying

Sun drying is one of mankind’s oldest preservation methods. Sun drying is a slow, gentle process that involves the exposure of a product to direct sunlight. This method is inexpensive and is a commonly used natural method of heat drying [90,91,92]. Opara et al. [9] investigated the vitamin C content and antimicrobial properties of fresh and sun-dried fractions of pomegranate fruit peel from locally grown (Oman pomegranate) and imported pomegranate (i.e., Indian red “Baghva”, Indian red “Ruby”, Indian white, and Egypt pomegranate) in Oman. The results showed that sun drying enhanced moisture loss and vitamin C retention and slightly enhanced the antimicrobial effects on *S. aureus* and *P. aeruginosa* by 3.77% and 15.23%, respectively. These data validated the idea that slow drying over a long duration enhances vitamin C retention in comparison with rapid high-temperature drying. Al-Rawahi et al. [85] examined the effects of four common postharvest drying treatments (freeze-drying, air drying, vacuum drying, and sun drying) on the phenolic content of pomegranate peels (cv. Helow). The sun-dried peels had phenolic contents comparable to those obtained using commercial drying methods at 3670, 1380, and 4080 mg gallic acid equivalent (GAE)/100 g dry peel solids using methanol, ethanol, and water for extraction, respectively. Contrary to this, El-Said et al. [18] noted that the total phenolic content of pomegranate peel extract for sun-dried peel ranged from 14.83 to 15.80 mg gallic acid/g in methanol extract and from 13.98 to 14.81 mg gallic acid/g in water extract.

Kumar et al. [86] standardized several drying techniques such as hot air drying, silica gel drying, microwave drying, air drying, and sun drying for different types of fruit peels for making potpourri. The study specifically tested acid lime peel, pomegranate peel, mandarin peel, sweet orange peel, and custard apple peel. The authors showed that sun and air drying times ranged from 3 to 5 days depending on the fruit peel type, and pomegranate peel had the longest drying period of 5 days. In addition, sun drying was slower compared to hot air and microwave drying techniques, which ranged from 16 to 29 h and from 5 to 9 min, respectively. Most vitamins and polyphenols are heat-sensitive compounds. Therefore, the low temperatures associated with sun drying may assist with the slow and gradual removal of moisture in the pomegranate peel compared to other drying techniques. Even though the sun drying technique may be suitable for small-scale production due to cost, the implementation of the sun drying method for pomegranate peels in large-scale production may negatively influence the safety and stability of the product. Consequently, the implementation of other drying methods may be required.

### 3.2. Microwave Drying

Microwave drying is fast becoming popular. Several researchers have used this method to process banana peels [93,94], orange peels [95,96], lemon peels [97], papaya peels [98], pitaya peels [99], and pomegranate peel waste [86,88,89], to name a few. For pomegranate peel processing using microwave drying, the study by Calín-Sánchez et al. [88] on cultivar Mollar de Elche showed that vacuum microwave drying at 240 W provided better results, giving punicalagin isomer contents (α- and β-PC) of 85.6 and 74.6 mg/g of DW, respectively, compared to convective predrying and other microwave drying levels studied (Table 4). However, this result was significantly lower compared to freeze-dried pomegranate peel for punicalagin isomer contents (α- and β-PC) and ellagic acid content, which were 113, 98.7, and 1.71 mg/g of DW, respectively. In addition, the total phenolic content followed a similar trend for fresh pomegranate peel at 125 mg gallic acid equivalent (GAE)/g, followed by freeze-drying at 118 mg gallic acid equivalent (GAE)/g and vacuum microwave drying at 111 mg gallic acid equivalent (GAE)/g. The rest of the drying levels and techniques significantly lowered the measured phenolic content.

Turrini et al. [89] tested the effect of using traditional oven drying (TD), single-mode drying (MH), hydrodiffusion drying, and gravity microwave oven drying (MHG) coupled with ultrasound extraction as green technologies for drying and subsequent valorisation of external pomegranate peels (cv. Wonderful and Acco). The study showed that microwave drying (both MH and MHG) significantly reduced the drying time from the 48 h reported for traditional oven drying at 40 °C to 5 min at ˂100 °C using microwave drying. However, when using a single-mode microwave oven dryer, the cv. Wonderful peel showed better results compared to Acco cultivar concerning drying efficiencies. It was suggested that the thickness of the exocarp of cv. Wonderful peel is an important consideration since the peculiar volumetric heating of MH is perhaps more effective if the material to be dried is thicker. In addition, the microwave hydrodiffusion gravity dryer allowed better recovery of the total phenolic compounds, ellagitannins, and radical scavenging abilities, which ranged 190–230 mg gallic acid equivalent (GAE)/100 mL, 106–110 µg/mL, and 480–560 mg ascorbic acid equivalent antioxidant capacity(AEAC)/100 mL, respectively, showing great potential for the production of high-value products (Table 4).

Similarly, reduced drying time for pomegranate peel was shown by Kumar et al. [86], who evaluated the efficacy of five drying methods (i.e., air drying, sun drying, silica gel drying, hot air oven drying, and microwave drying) for four fruit peel types, including sweet orange peel, mandarin peel, custard apple skin, and pomegranate peel. The pomegranate peel was indicated to have the highest dry weight and lowest moisture loss compared to the other fruit peels at 34.67 g and 65.33%, respectively, using microwave drying. Furthermore, microwave drying was the quickest drying method for all fruit peel types tested, with samples drying within 5 to 9 min compared to all tested drying techniques, which took many hours, ranging from 16 to 29 h for hot air drying and even days for the other drying techniques to dry the peels effectively. Drying using microwave energy is a cost-efficient and a faster alternative to other drying methods, plus it offers a modern way to preserve the quality (chemical, sensory, and nutritional) of dried pomegranate peel products. Microwave drying, to our knowledge, is not widely used in the food industry, especially for fruit peel waste preservation; thus, more research on the application of this technique is still necessary.

### 3.3. Freeze-Drying

Freeze-drying, also known as lyophilisation, involves dehydration by sublimation of a frozen sample and desorption under vacuum conditions [100]. The solid state of water during freeze-drying preserves the primary structure, aroma, and flavour of the product, causing very low reductions in volume [100]. Mphahlele et al. [34] determined the effects of drying on the bioactive compounds and antioxidant, antibacterial, and antityrosinase activities of pomegranate peel (cv. Wonderful) using freeze-drying, confirming that overall the freeze-dried peel had the lowest residual moisture. Furthermore, freeze-dried peel had the highest rutin, catechin, epicatechin, and hesperidin contents at 4666.03, 674.51, 70.56, and 16.45 mg/kg DM, respectively. Furthermore, freeze-drying gave the highest total phenolic content (TPC), total tannin concentration (TTC), and total flavonoid concentration (TFC) values, which were between 3- and 5-fold higher in freeze-dried peel than the oven-dried peel at all temperatures (40, 50, and 60 °C). Calín-Sánchez et al. [88] observed the highest content of punicalagin isomers (α-PC and β-PC) in freeze-dried peels at 113 and 98.7 mg/g of DW, respectively, compared to the vacuum microwave drying method (Table 4).

Freeze-dried pomegranate (cv. Helow) peels were shown to have phenolic contents comparable to those of fresh pomegranate peels (i.e., 4900 and 4010 mg gallic acid equivalent (GAE)/100 g dry peel solids) [85]. John et al. [87] explored the possible effects of processing pomegranate peel using three drying conditions (freeze-drying, oven drying at 50 °C, and ambient drying at ~25 °C) and the subsequent impacts of on the bioactive ellagitannins (punicalins, punicalagins, and ellagic acid) in the peel and other fruit fractions. The observations of the study indicated that the total phenolic content varied between 80 and 96 mg/g gallic acid equivalents in the peel, with the highest amounts occurring in the freeze-dried peel, whilst other drying techniques showed a 15% decline in the total phenolic content. Moreover, freeze-dried pomegranate peel showed higher α-punicalin and β-punicalin contents at 0.8 and 1.6 mg/g, respectively. High punicalagin (~38.6–50.3 mg/g) and ellagic acid (~2.8–3.2 mg) contents were evident in the peels and inner membrane fractions of all three dried samples. The 2,2-diphenyl-1-picrylhydrazy (DPPH) content followed a similar trend as the total phenolic content, with the highest radical scavenging activity being observed in freeze-dried samples, with 10–13% decreases observed with the other two drying conditions (Table 4).

Freeze-drying preserves the primary structure, aroma, and flavour of a product, with a very low reduction in the volume [100]. This could explain the higher phenolic content recorded for pomegranate peel compared to other drying techniques. Furthermore, the freeze-drying technique favours preservation of heat-sensitive phytochemicals, as temperatures are extremely low during the dehydration process. However, freeze-drying is energy-intensive, as it requires long drying time, and thus is expensive. For this reason, freeze-drying may not be a practical or cost-effective option for large-scale production of pomegranate peel waste products.

### 3.4. Vacuum Drying

Vacuum drying can be a useful technique for solid products that are heat-sensitive. This method involves the dehydration of wet or damp material in a chamber at low pressure and temperature, thus creating a vacuum. The decrease in pressure causes the expansion and escape of gas occluded into pores. Vacuum pressures of 50 to 100 mbar are used in the system to achieve the desired outcome [84]. Several researchers have used vacuum drying for fruit peel waste processing, such as for banana fruit peels [93,101], gac fruit peels [102], kinnow fruit peels [103], mango fruit peels [104], orange fruit peels [95], and various other fruit peel waste products. However, there is almost no scientific information in relation to pomegranate peel studies, as only a single study by Al-Rawahi et al. [85] was found in our literature search. In that paper, the researchers determined the effects of four common drying treatments (freeze-drying, air drying, vacuum drying, and sun drying) on the phenolic contents of pomegranate peels, showing that vacuum drying significantly reduced the phenolic content of the pomegranate peel compared to the other tested methods. Furthermore, vacuum drying did not show any trends related to temperature (40, 60, and 90 °C), giving values of 1200 and 5330 mg gallic acid equivalent (GAE)/100 g dry peel solids with extraction in ethanol at 40 °C and in water at 90 °C, respectively (Table 4). The technique is energy- and cost-effective, with short drying times at low temperatures that do not damage the product. Another advantage of applying this method is that it does not cause corrosion issues and is environmentally friendly. Unfortunately, for large-scale production, it may not be as cost-effective. This may be the reason why few studies exist illustrating the application of the method on pomegranate peel.

### 3.5. Oven Drying

Oven drying is another pre-extraction method that uses thermal energy to remove moisture from the sample material. This sample preparation method is considered one of the easiest and most rapid thermal processing methods for preserving phytochemicals. Opara et al. [9] reported that pomegranate peels of the white variety lost a moderately higher amount of moisture at 64.49% with sun drying versus 68.73% with oven drying, as compared to other cultivars such as Bhagva, Ruby, Egypt, and Oman. The authors attributed this to differences in the fruit mass and peel structure. Mphahlele et al. [34] showed that drying pomegranate (cv. Wonderful) peel at 50 °C gave the highest inhibitory activity, with minimum inhibitory concentration values of 0.10 mg/mL against Gram-positive bacteria (*Staphylococcus aureus* and *Bacillus subtilis*). Furthermore, oven drying at 60 °C resulted in a high punicalin concentration of 888.04 mg/kg DM, while *p*-coumaric acid was found at 0.57 mg/kg DM (Table 4). However, at 40 °C *p*-coumaric acid was detected at 0.45 mg/kg DM, plus the vitamin C concentration was found to be highest at 31.19 μg ascorbic acid equivalent (AAE)/g DM.

El-Said et al. [18] studied the use of pomegranate peel extract in the manufacture of stirred yoghurt and determined the antioxidant and organoleptic properties. The total phenolic contents of the pomegranate peel subjected to oven drying ranged from 16.00 to 17.78 mg gallic acid/g in methanol extract and from 14.23 to 16.34 mg gallic acid/g in water extract when kept at 40 °C for 48 h, as compared to the values for solar dried peel kept at 50 °C for 2 h, which ranged from 14.83 to 15.80 mg gallic acid/g in methanol extract and from 13.98 to 14.81 mg gallic acid/g in water extract. The radical scavenging activity was detected in the following order: whole > inside > outside peel extracts, regardless of whether oven- or solar-dried for both extracts (methanol and water extracts). Other studies detailing users of this technology are shown in Table 4. The oven drying technique for pomegranate peel has been shown to improve the quality of the dried pomegranate peel material. It is easily accessible and economical. However, it may reduce the integrity of the product from its original state due to the use of high temperatures and prolonged drying periods during the process.

## 4. Effects of Extraction Methods

The secondary metabolites in the pomegranate peel are affected by various horticultural factors, such as the fruit cultivar, growing region, agro-climate, maturity, cultivation practice, processing factors (drying and extraction), and storage conditions [2,8,10,64]. Such variations influence the physical and chemical makeup of the pomegranate fruit peel, affecting the quality of the fruit peel products. Finding novel ways to optimise the extraction of these phytochemicals and using pretreatment to reduce the loss of these heat-sensitive phytochemicals is necessary in the competitive pharmaceutical and nutraceutical industries.

### 4.1. Solvent Extraction

The extraction of phytochemicals from pomegranate peel is beneficial, especially the isolation of antioxidant compounds, which have great value in the health and cosmetic industries. Water, ethanol, and their combinations are most suitable for products intended for human consumption, representing the best possible food grade solvents for commercial extraction of phenolics in pomegranate peel [67,86,104,105,106,107]. Al-Rawahi et al. [85] evaluated the effects of different solvents (i.e., water, methanol, and ethanol) used for the extraction of phenolic compounds from pomegranate (cv. Helow) peel. The aqueous peel extract was shown to have a higher capacity for extraction and higher total phenolic contents than methanol and ethanol pomegranate peel extracts, with values of 8460, 5990, and 4530 mg gallic acid equivalent (GAE)/100 g dry peel solids (DM) for water, methanol, and ethanol, respectively. Similarly, finding the most effective solvent (i.e., water, ethanol, and methanol), either alone or in combination, for the extraction of the potent antioxidant, phenolic, and antibacterial compounds from pomegranate (cv. Ganesh) peel was the main objective of the work by Malviya et al. [106]. The authors indicated that the highest total phenolic content was observed using aqueous extract at 438.3 ± 14.15 mg/g. Water, ethanol peel extracts, and their combinations are, however, limited to very low antioxidant yields compared to other solvents, such as methanol.

Methanolic pomegranate peel extracts have been proven to give superior results over other solvent extracts [108,109,110,111]. Wang et al. [109] considered different solid–solvent extractions of antioxidants from the pomegranate (cv. Wonderful) marc peel (PMP) and further elucidated how different solvents (water, methanol, ethanol, acetone, and ethyl acetate), temperatures, and solvent–solid ratios affect the extraction of antioxidant compounds. The highest extract and phenolic yields were recorded for the methanolic peel extract at 46.51 g dried extract/100 g pomegranate marc peel (PMP) and 8.26%, respectively. Similarly, Shiban et al. [112] evaluated the antioxidant activity of pomegranate (Yemeni varieties) peel extracts using in vitro methods from three solvent peel extracts (80% methanol, distilled water, and diethyl ether extracts). They showed that the 80% methanolic peel extract had the highest extraction yield, total phenolic content, and total flavonoid content at 45.4%, 274 mg gallic acid equivalent (GAE)/g (27.4%), and 56.4 mg flavonoids rutin equivalent (RE)/g, respectively.

Fawole et al. [8] evaluated the antibacterial, antioxidant, and tyrosinase inhibition activities of methanolic extracts from the peels of seven commercially grown pomegranate varieties (cvs. Ganesh, Molla de Elche, Arakta, Bhagwa, Wonderful, Herskawitz, Ganesh, and Ruby) using in vitro assays. The tested 80% methanolic peel extract gave high total phenolic content values compared to aqueous extracts. The highest total phenolic content was identified as that generated from the Ganesh cultivar at 295.5 mg/g dry extract. Kaur et al. [113] investigated the antimicrobial and antioxidant potential of pomegranate peel using hexane, dichloromethane, ethyl acetate, and methanol as solvents. They further emphasised the superiority of methanol extracts, as they excelled far above the other tested solvents. In fact, the highest exact yield of 37.85% was obtained for the methanol extract, while the hexane gave the lowest extract yield of 2.1%. Furthermore, the methanolic extract showed higher amounts of 5-hydroxymethylfurfural as a major compound at 60.11%, a higher radical scavenging activity (RSA) value of 75.36% at 100µg/mL, and a minimum value of 41.20% at 10µg/mL.

Numerous works have studied methanolic extraction of pomegranate peel [108,114,115,116] (Table 5). Other researchers have suggested that a mixture of solvents for pomegranate peel extraction is more beneficial than individual solvents [116,117]. Extraction of phytochemicals in pomegranate peel is beneficial, especially for isolating antioxidant compounds, which have great value in the health and cosmetic industries. However, efficient methods of extraction are important for designing efficient extraction processes [19,109]. Usually, the selection of an appropriate extraction technique and solvent type is dependent on the type of compound to be extracted, safety issues (especially for the food industry and for preparations of ingredients for human skin), and the potential up-scaling of the technique. Normally, the extraction solvent, temperature, solid–liquid ratio, and particle size are influential parameters in the extraction process, with various solvents affecting the antioxidant activity, because the quantity profiles of pomegranate peel extract are largely dependent on the type of extraction solvents used [107,116]. Therefore, it is necessary to optimise the extraction conditions associated with solvent extraction to obtain maximum yields, such as the solvent ratio, the particle size of the pomegranate peel powder, the extraction time and temperature, and the solvent–solid ratio.

### 4.2. Supercritical Fluid Extraction (SFE)

Supercritical extraction technologies have been used extensively in the past to extract target compounds from a variety of matrices at commercial and laboratory scales. For quantification purposes, this technique is often used for the recovery of the total target analyte and can grant optimal conditions for bioactive compound recovery with the aim of commercial extraction. The technique is a pressurised extraction method that reduces waste solvent production. Its high standard of recovery of pure compounds makes it a suitable match for the extraction of phytochemicals in the pomegranate peel.

Several researchers have used this method for pomegranate peel extraction (Table 5). Ara and Raofie [118] extracted essential oil compounds from pomegranate peel using supercritical fluid extraction, whereby the results were compared with those obtained using the hydrodistillation technique. Supercritical fluid extraction led to a higher extract yield of 1.18% (*v*/*w*), whereas a value of 0.21% (*v*/*w*) was noted for hydrodistillation. Bustamante et al. [119] optimised phenolic compound and oil extraction from pomegranate peel with supercritical CO_2_ and peel extracts using a supercritical carbon dioxide extraction process, which gave a high punicalagin content, total phenolic content, and antioxidant activity with values of 9.7%, 10.01 mg gallic acid equivalent (GAE)/g, and 99.4 lg Trolox equivalent per g, respectively, once optimal conditions were determined. Similarly, high phenolic content recovery was noted in a study by Mushtaq et al. [120], who utilised the maceration power of different enzymes to develop an effective, quick, and green extraction protocol for the optimum recovery of phenolic antioxidants from pomegranate peel. They confirmed that enzyme-assisted supercritical fluid extraction (EASCFE) doubled the recovery of crude extracts, with increased levels of total phenolic content reported (301.53 mg gallic acid equivalent (GAE)/g). In addition, numerous phenolic compounds were present in high amounts, such as vanillic acid contents ranging from 65.87–108.36 µg/g, in enzyme-assisted solvent extraction and enzyme-assisted supercritical fluid extraction (Table 5).

Several published papers have shown that pomegranate peel contains bound and free phenolics [63,121]. Therefore, there is a need to optimise the recovery of these phenolics, which have been proven to be beneficial for health and for the industry [63,121]. Furthermore, carbon dioxide is a safe, food-grade product that is widely available at a relatively low cost and with high purity [120,122]. However, the disadvantage is that carbon dioxide is relatively apolar, requiring the addition of a co-solvent such as methanol or ethanol for polar analyte extraction [120,123]. This, however, does not assist the extraction of bound phenolics. Thus, a pretreatment process before extraction may be used to improve or release the bound phenolics by breaking down the cellulosic composite structure of the cell wall [120]. Supercritical extraction technology is a green process for extraction and isolation of high-value natural products and phytochemicals. The technique is relatively safe and does not alter the structures of heat- and oxidation-sensitive compounds [120,123].

**Table 5:** The effect of extraction techniques on the phytochemical and nutritional properties of the pomegranate peel quality.

Extraction Technique	Cultivar	Country	Phytochemical and Nutritional Properties	Key Findings	Reference
Solvent extraction 80% (*v*/*v*) methanol and distilled water (aqueous)	Ganesh, Molla de Elche, Arakta, Bhagwa, Wonderful, Herskawitz, Ganesh, and Ruby	South Africa	Total flavonoid content (TFC), gallotannin content (GTC), total anthocyanin content (TAC), catechin, epicatechin, ellagic acid, and gallic acid	High amounts of phenolic compounds were found in peel extracts, with the highest total phenolic content (TPC) of 295.5 mg/g dry extract found in Ganesh and the lowest in cultivar Molla de Elche at 179.3 mg/g dry extract. Catechin, epicatechin, ellagic acid, and gallic acid were found in all cultivars, with ellagic acid being the most abundant. None of the aqueous extracts exhibited good antibacterial activity at the highest screening concentration (>12.5 mg/mL).	[8]
Solvent extraction water, methanol, and ethanol	Helow	Sultanate of Oman	Total phenolic content (TPC)	Water extracted the highest amount of total phenolic content (TPC), followed by methanol and ethanol. Fresh peel extracts contained 5990, 4530, and 8460 mg gallic acid equivalent (GAE)/100 g dry peel solids (recorded as dry mass (DM)) for methanol, ethanol, and water extracts, respectively.	[85]
Solvent extraction Aqueous and methanolic extracts	Unknown	Egypt	Total phenolic content (TPC) and total flavonoid content (TFC)	Methanol extracts showed highest total phenolic content (TPC) and total flavonoid content (TFC) amounts, regardless of the mode of drying used—methanolic oven-dried peel extracts ranged from 16.00 to 17.78 mg gallic acid/g. The total phenolic content (TPC) of aqueous peel ranged from 14.23 to 16.34 mg gallic acid/g with the same drying technique. Total flavonoid content (TFC) values ranged from 6.99 to 7.98 and 6.54 to 6.86 mg rutin equivalent (RE)/g for methanolic and aqueous oven-dried extracts.	[18]
Solvent extraction Water, methanol (MeOH), ethanol, acetone, and ethyl acetate (EtOAc) extraction (15:1 (*w*/*w*) at 40 °C for 4 h)	Wonderful	USA	Extract yield, total phenolic content (TPC), proanthocyanidins, and total flavonoid content (TFC)	Highest extract yield in methanol (MeOH) at 46.51 ± 0.86, followed by water (H_2_O) at 43.19 ± 2.24. The lowest was ethyl acetate at 0.88 ± 0.08 g dried extract/100 g pomegranate marc peel(PMP). MeOH followed by H_2_O had the highest phytochemical yields, with total phenolic content (TPC) values of 5.90% and 8.26%, respectively. The concentrations of total phenolic content (TPC), pro-anthocyanidins, and (total flavonoid content (TFC) were highest using ethyl acetate at ≤20%, followed by methanol (MeOH) at 18%, distilled water (H_2_O) at 14%, and ethanol at 9%.	[109]
Solvent extraction 80% methanol (MeOH), distilled water (H_2_O), and diethyl ether extracts	Yemeni varieties	Saudi Arabia	Extract yield, total phenolic content (TPC), total flavonoid content (TFC), and ascorbic acid	80% methanol (MeOH) extract had the highest yields for total phenolic content (TPC) and total flavonoid content (TFC) at 45.4 ± 5.3% and 274 ± 17 mg gallic acid equivalent (GAE)/g (27.4%) and 56.4 ± 2.7 mg for flavonoids rutin equivalent (RE)/g. Ascorbic acid (AA) was only present in small amounts (2 mg/g), and thus it was unlikely to substantially contribute to the antioxidant activity of methanol (MeOH).	[112]
Solvent extraction MeOH at 30 °C for 4 h	Ganesh	India	Extract yield, total phenolic content (TPC), gallic acid, and ellagic acid	Methanol (MeOH) extract had the highest yield at 10.38 ± 0.89% weight for weight (*w*/*w*). The total phenolic content of the extract [(+)-catechin equivalent] was found to be 42% weight for weight (*w*/*w*). The high performance liquid chromatography (HPLC) pattern of the methanol (MeOH) extract of peel showed the presence of gallic acid and ellagic acid as the major components. Methanol (MeOH) pomegranate peel extract was reported to enhance or maintain the activity of hepatic enzymes.	[114]
Solvent extraction Water, ethanol (50, 70 and 100% ethanol), and methanol (100%)	Ganesh	India	Extract yield, total phenolic content (TPC)	The highest yield was reported using 50% ethanol at 16.3 ± 1.99%, while aqueous extract had the highest total phenolic content (TPC) at 438.3 mg/g. Higher 2,2-diphenyl-1-picrylhydrazy (DPPH) and 2,2′-azino-bis (3-ethylbenzthiazoline-6-sulfonic acid) (ABTS) inhibition was reported using methanol and 70% ethanol at 79.5 ± 6.5 and 94.6 ± 6.10, respectively.	[106]
Solvent extraction Aqueous, ethanol, chloroform, acetone, and petroleum ether	Unknown	India	Extract yield, tannins, saponins, flavonoids, quinones, cardiac glycosides, terpenoids, phenol, steroid, coumarins, and alkaloids	The optimum tannin yield was reported at 87.3 mg tannic acid equivalent (TAE)/g using ethanol extract. Tannins, saponins, flavonoids, quinones, cardiac glycosides, terpenoids, phenol, steroid, coumarins, and alkaloids. Highest antioxidant activity was reported at 94.5% using ethanol extract.	[124]
Soxhlet extraction (solvent extraction) Hexane, dichloromethane, ethyl acetate, and methanol	Unknown	India	Extract yield, 5-hydroxy- methylfurfural, furan-2,5- dicarbaldehyde	The highest exact yield was obtained at 37.85% using methanol and the lowest at 2.1% using hexane. Methanolic extract showed higher 5-hydroxymethylfurfural as a major compound at 60.11%, higher radical scavenging activity (RSA) of 75.36% at 100µg/mL, and a minimum value of 41.20% at 10µg/mL. Furan-2,5-dicarbaldehyde was reported with high radical scavenging activity (RSA) of 70.39% at 100µg/mL and 39.03% at 10µg/mL.	[113]
Solvent extraction Methanol, ethanol, acetone, chloroform, and ethyl acetate	Unknown	India	Extract yield, total phenolic content (TPC), methyl gallate, tocopherol, quercitin glucoside, polymeric flavonol, maclurin-3-c-(2-o-galloyl)-β-d-glucoside, epicatechin, and quercitin pentoside	Methanol had the highest yield at 23.56%, while the lowest yield was 2.2% using ethyl acetate. Similarly, the highest total phenolic content (TPC) value was found in methanol and the lowest in ethyl acetate peel extracts at 78.92 and 1.30 mg/gm gallic acid equivalent (GAE), respectively.	[115]
Solvent extraction ethyl acetate (EtOAc), methanol, and water	Ganesh	India	Moisture content (%), extract yield, total phenolic content (TPC), gallic acid	Methanolic peel extracts had the highest extract yield at 9.38% weight for weight (*w*/*w)*, followed by water at 7.53% weight for weight (*w*/*w)* and ethyl acetate (EtOAc) at 1.04% weight for weight (*w*/*w)*. Methanol (MeOH) extract had highest total phenolic content (TPC) at 44.0% weight for weight *(w*/*w)*, while lowest was found in aqueous extract at 3.00% weight for weight (*w*/*w*).	[108]
Solvent extraction Ethanol, methanol, acetone, and combinations	Unknown	China	Extract yield, total phenolic content (TPC), flavonoids, pro-athocyanidins, and ascorbic acid	Peel combination solvent extract had higher ferric reducing antioxidant power (FRAP) value (approximately 4.5 mmol/L) than those obtained using individual solvents. Extract yield, total phenolic content (TPC), flavonoids, proanthocyanidins, and ascorbic acid of peel extract were reported at 31.5 ± 3.1%, 249.4 ± 17.2 mg/g, 59.1 ± 4.8 mg/g, 10.9 ± 0.5 mg/g, and 0.99 ± 0.02 mg/g, respectively.	[125]
Solvent extraction ethyl acetate (EtOAc), acetone, methanol (MeOH), and water	Unknown	India	Extract yield and Total phenolic content (TPC)	Methanol (MeOH) extract had highest total phenolic content (TPC) yields at 9.4% and 460 mg/g, respectively.	[116]
Solvent and ultrasound-assisted solvent extraction methods (acetone, methanol (MeOH), ethanol (EtOH), water (H_2_O), ethyl acetate) and supercritical fluid extraction (SFE) of (CO_2_)	Poost Syah	Iran	Phenolic compounds, punicalagin	Punicalagin content range was 0.32–0.84 g/100 g dryweight (DW) using supercritical fluid extraction (SFE). Acetone with sonication produced the highest phenolic compound contents (in either solvent or ultrasound-assisted solvent extraction methods, 40.0 and 35.0% for sonication and solvent extraction, respectively; lowest values were found in ethyl acetate extracts (0.2 and 0.2%)). The ethyl acetate extract and extract of modified supercritical fluid extraction carbon dioxide (SFE CO_2_) had similar but comparatively small extraction yields.	[126]
Supercritical fluid extraction (SFE) and hydrodistillation	Malas	Iran	Over 76 essential oils including oleic acid, palmitic acid, and (−)-borneol	Over 76 essential oils were identified using gas chromatography mass spectrometry (GCMS), with oleic acid, palmitic acid, and (−)-borneol being the major compounds in both extracts. The optimum extraction yields were 1.18% weight for weight (*w*/*w*) for supercritical fluid extraction (SFE) and 0.21% volume per weight (*v*/*w*) for hydrodistillation.	[118]
Supercritical carbon dioxide (SC-CO_2_) using a Box–Behnken design	Wonderful	Chile	Extract yield, total phenolic content (TPC), phenolic compounds (punicalagin (PU) and punicic acid (PA))	Extract yields ranged from 0.2% to 8.5%, with the highest yields at 200 and 300 bar, 40–50 °C, and 20% cosolvent. Punicalagin (PU) contents of 0.4–9.5% with optimal extraction conditions of 400 bar, 43 °C, and 20% ethanol. Once the peel extract was extracted under optimal conditions, the extract had a punicalagin (PU) content of 9.7%, total phenolic content (TPC) of 10.01 mg gallic acid equivalent (GAE) per g, and an antioxidant activity of 99.4 (lg Trolox equivalent per g). The punicalagin EE ranged from 35.1% to 72.4%.	[119]
Enzyme-assisted supercritical fluid extraction (EASCFE) and enzyme-assisted solvent extraction (EASE)	Unknown	Pakistan	Extract yield, Total phenolic content (TPC), phenolic acids: vanillic acid, ferulic, syringic, sinapic acid	The highest extract yield of 65.89% was obtained using enzyme-assisted solvent extraction (EASE). Enzyme-assisted supercritical fluid extraction (EASCFE) had the highest total phenolic content (TPC) at 301.53 mg gallic acid equivalent (GAE)/g pretreated under optimised conditions involving cocktail enzyme concentration (3.8%) at 49 °C and pH of 6.7 for 85 min. Numerous phenolic compounds were present in high amounts, such as vanillic acid ranging at 65.87 and 108.36 µg/g in enzyme-assisted solvent extraction (EASE) and enzyme-assisted supercritical fluid extraction (EASCFE), respectively. The *p*-coumaric acid (0.12–14.87 µg/g), gallic acid (0.041–0.37 µg/g), caffeic acid (3.88–75.19 µg/g), ferulic acid (0.15–8.84µg/g), syringic acid (15.17–88.24 µg/g), and sinapic acid (2.13–3.58 µg/g) were assayed after enzyme-assisted solvent extraction (EASE) and enzyme-assisted supercritical fluid extraction (EASCFE).	[120]
Ultrasound-assisted extraction (UAE) Water	Sishe Kape-Ferdos	Iran	Moisture content, extract yield, and total phenolic content (TPC)	The moisture content was reported at 46.62 wet basis (%wb). The optimal conditions based on both individual and combinations of all process variables were ultrasonic amplitude (UA) of 60% and ultrasonic exposure time (UET) of 6.2 min. With these optimum conditions, the predicted maximum yield and total phenolic content (TPC) values were 13.1% and 42.2 mg gallic acid (GA)/g, respectively.	[127]
Ultrasound-assisted enzymatic extraction	Dalim	India	Total phenolic content (TPC) and total flavonoid content (TFC)	The optimum conditions for maximum extractability were an ultrasonication time of 41.45 min, enzyme concentration of 1.32 mL/100 mL, incubation time of 1.821 h, and incubation temperature of 44.85 °C. The total phenolic content (TPC), total flavonoid content (TFC), and radical scavenging activity (RSA) values in optimised conditions were 19.77 mg gallic acid equivalent (GAE)/g, 17.97 mg quercetin equivalent (QE)/g, and 74.213%, respectively.	[128]
Ultrasound-assisted extraction Ethanol	Unknown	Serbia	Total phenolic content (TPC), phenolic compounds: ellagic acid, gallic acid, punicalagin, and punicalin	Optimal extraction process conditions were as follows: extraction time of 25 min, ethanol concentration of 59%, solid-to-solvent ratio of 1:44, and extraction temperature of 80 °C. Total phenolic content (TPC) values in extracts obtained using ultrasound-assisted extraction (UAE) technique varied between 81.61 and 190.94 mg gallic acid equivalent (GAE)/g dry weight (DW). Ellagic acid, gallic acid, punicalin, and punicalagin content ranges were 4.05–12.54, 1.13–3.58, 28.38–65.67 and 7.04–35.05 mg/g dry weight (DW), respectively.	[129]
Ultrasound-assisted extraction Sunflower and soy oils	Unknown	Greece	Extract yield and carotenoids	The optimum extraction yield was about 0.3255 mg carotenoids/100 g of dry peels. The levels of extraction using the green solvents and ultrasound were about 85.7 and 93.8% of the total carotenoids present in the waste material, respectively. The highest concentrations obtained were 0.6134 and 0.6715 mg carotenoids/100 g of dry peels using sunflower oil and soy oils, respectively.	[130]
Pulsed ultrasound-assisted extraction (PUAE)	Malas	Iran	Extract yield, punicalagin, ellagic acid, gallic acid, and hydroxybenzoic acids	The application of 10 min extraction under the pulsed mode also resulted in a high yield (41.6%). Punicalagin contents (α and β) varied from 128.02 to 146.61 mg/g, with the highest content obtained at an intensity level of 105 W/cm^2^ and duty cycle of 50% for a short time (10 min). Ellagic acid content varied from 10.12 to 22.53 mg/g with different pulsed ultrasound-assisted extraction (PUAE) conditions. High performance liquid chromatography (HPLC) analysis revealed that punicalagin, ellagic acid, and gallic acid constitute almost 168.55 mg/g of the peel extract.	[131]
Ultrasound-assisted extraction	Unknown	India	Extract yield of pectin	Optimal conditions were solid liquid (SL) ratio of 1:18 g/mL, pH of 1.3, extraction time of 29 min, and extraction temperature of 62 °C. Under these conditions, the experimental yield of pectin was reported at 23.87%, close to the predicted value (23.92%).	[132]
Ultrasound-assisted polysaccharide extraction	Unknown	China	Polysaccharide yield	Optimum extraction parameters were as follows: ratio of water-to-raw material of 24 mL/g, ultrasonic power of 148 W, extraction time and temperature of 63 min and 55 °C, respectively. Under these conditions, the polysaccharide yield was 13.658 ± 0.133%, similar to the predicted value of 13.787%.	[133]
Ultrasound-assisted extraction	Unknown	Greece	Extract yield	Direct ultrasound-assisted extraction of total phenolics gave a maximum yield of 13.85% (g gallic acid equivalent (GAE)/100 g of dry peels) with an extraction time of 10 min. The extraction yield increased with increasing extraction temperature from 25 to 35 °C, with amplitude levels up to around 40%, solvent/peel ratios of up to 33:1, and decreasing pulse duration/pulse interval ratios	[134]
Ultrasound-assisted extraction Ethanol–water mixture	Unknown	Iran	Extract yield, total phenolic content (TPC)	The extraction yield with water–ethanol as the solvent (45.4%) was four times better than the values for water, which were 11–14%.The extraction yield with water–ethanol as the solvent (45.4%) was also better than methanol (29–35.5%). Total phenolic content (TPC) varied from 5506.42 to 8923.24 mg gallic acid equivalent/100 g of dry weight. Optimal conditions were 70% ethanol–water mixture as the solvent, temperature of 60 °C, and extraction time of 30 min.	[135]
Ultrasonic-assisted extraction	Bhagwa	India	Extract yield, total phenolic content (TPC), total tannins (TE), phenolic compounds: gallic acid, ellagic acid, and punicalagin (α and β)	Optimum process conditions of 15.12 min (extraction time) and amplitude of 30% gave the highest yield, total phenolic content (TPC), and total tannins (TE) values of 42.45%, 354.67 mg gallic acid equivalent (GAE)/g, and 348.0 g TAE/g, respectively. High performance liquid chromatography (HPLC) analysis revealed gallic acid, ellagic acid, and punicalagin (α and β) as the major ellagitannin compounds at 0.96, 7.58, and 163.52 mg/g, respectively.	[136]
Ultrasound-assisted extractions in continuous (CUAE) and pulsed modes (PUAE) compared with convectional extraction (CE)	Wonderful	USA	Total phenolic yield (%)	High antioxidant yield (14.8%) was achieved at an intensity of 59.2 W/cm^2^ and treatment time of 60 min for ultrasound-assisted extractions in continuous (CUAE). 2,2-diphenyl-1-picrylhydrazy (DPPH) scavenging activities of 5.5 g/g at an extraction time of 60 min, temperature of 25 ± 2 °C, and water/peel ratio of 50:1 *w*/*w* elevated the antioxidant yield by 24% and decreased the extraction time by 90% compared to convectional extraction (CE). An intensity level of 59.2 W/cm^2^, pulse duration of 5, and interval of 5 s gave an antioxidant yield of 14.5%. Pulsed ultrasound-assisted extraction (PUAE) elevated the antioxidant yield by 22% and decreased the extraction time by 87% in comparison to convectional extraction (CE). It also conserved 50% more electrical energy than ultrasound-assisted extractions in continuous (CUAE). Pulsed ultrasound-assisted extraction (PUAE) was superior to ultrasound-assisted extractions in continuous (CUAE).	[137]
Microwave-assisted extraction (MAE) Used water	Unknown	China	Phenolic yield	The average experimental phenolic yield under the optimum conditions was found to be 210.36 ± 2.85 mg gallic acid equivalent (GAE)/g, similar to the predicted value of 214.46 mg GAE/g. It was found that the extract was an effective scavenger in quenching 2,2-diphenyl-1-picrylhydrazy (DPPH) radicals, with an inhibitory concentration (IC_50_) of 14.53 µg/mL.	[138]
Microwave-assisted extraction (MAE) Ethanol	Unknown	China	Flavonoids content and yield of flavonoids	A maximum extraction yield of 4.26% was achieved at an ethanol concentration of 60%, solvent-to-material ratio of 40:1, and microwave-assisted extraction (MAE) time of 3 min. The extract exhibited a strong 2,2-diphenyl-1-picrylhydrazy (DPPH) radical scavenging ability, with an inhibitory concentration (IC_50_) value of 0.187 mg/mL.	[139]
Microwave-assisted extraction, ultra-assisted extraction, conventional solid–liquid extraction (maceration and decoction)	Unknown	Mexico	Total phenolic content (TPC), phenolic compounds	The microwave extraction method obtained the highest total phenolic content (TPC) at 18.92 mg gallic acid equivalent (GAE)/g, and 14 compounds were identified using both extraction methods. Both extraction methods showed important differences. Cinnamic acid (*m*/*z* 146.9279) and granatin B (Galloyl-HHDP-DHHDP-hexoside) (*m*/*z* 950.7491) were only reported in decoction peel extracts. Secoisolariciresinol di-O-glucoside (*m*/*z* 540.8878) and pedunculagin I (*m*/*z* 782.8182) were reported in microwave-assisted extraction (MAE) peel extracts.	[140]
Pressurised liquid extraction Deionised water	Izmir 8, Izmir 10, Izmir 16, Izmir 23, Izmir 26, Izmir 1264, Izmir 1479, Izmir 1499, Izmir 1513	Turkey	Extract yield, total phenolic content (TPC), total flavonoid content (TFC), condensed tannins (CT), hydrolysable tannins (HT), punicalagin (α and β), gallic acid and ellagic acid	Extract yields of 43.3, 46.5, and 16.7% reported for pressurised water extraction (PWE), methanol, and water extraction, respectively. Total phenolic content (TPC) values of 264.3, 258.2 and 80.5 mg tannic acid equivalent (TAE)/g reported using pressurised water (PWE), methanol, and water extraction, respectively. Total flavonoid content (TFC) values of 13.0, 18.1, and 6.2 mg catechin equivalent (CE)/g reported using pressurised water extraction (PWE), methanol, and water extraction, respectively. Hydrolysable tannins (HT) ranged from 262.70 to 82.6 mg tannic acid equivalent (TAE)/g (water extraction). Condensed tannins (CT) values of 9.5, 9.2, and 3.7 mg catechin equivalent (CE)/g dry weight (DW) using PWE, methanol, and water extraction, respectively. Punicalagin content (α and β), ellagic acid, and gallic acid values of 116.6, 1.25, and 1.43 mg/g, respectively.	[141]
Ultrasound-assisted pressurised liquid extraction (UAPLE) Water, ethanol + water 30, 50, and 70% *v*/*v*)	Wonderful	USA	Extract yield, α- and β-punicalagin, ellagic acid hexoside. and ellagic acid. Ellagic acid pentoside, ellagic acid deoxyhexoside, pedunculagin I, and unidentified compounds	Ultrasound extraction was reported to have a higher influence on the extraction yields when utilising large particles measuring 1.05 mm and intermediate ultrasound power of 480–640 W at the generator or 23.1–30.8 W at the tip of the probe. Using ultra-high performance liquid chromatography tandem mass spectrometry (UHPLC-MS/MS), a total of 24 different compounds were detected. Using a larger peel particle size of 1.05 mm, water extraction, extraction temperature of 70 °C, ultrasound power of 480 W, and 3 cycles, am enhanced phenolic recovery yield of 61.72 ± 7.70 mg/g of phenolic compounds was achieved from the pomegranate peel.	[142]
High-pressure assisted extraction	Unknown	Portugal	Extract yield, individual phenolics, tannins, and anthocyanins	Increase of the extraction time and pressure and decrease of ethanol concentration, as well as enhanced extract yields (only until a pressure of 382 MPa and ethanol concentration of 36%). The optimum extraction conditions were comparable for all compounds, excluding anthocyanins, which varied between 356 and 600 MPa, with an extraction time of 30 min and an ethanol concentration of 56%. The optimum conditions were reported at an extraction time of 30 min, pressure of 492 MPa, and an extraction concentration of 37%.	[143]

**Abbreviations:** Total flavonoid content (TFC), gallotannin content (GTC), total anthocyanin content (TAC), gallic acid equivalent (GAE), rutin equivalent (RE), dry mass (DM)), wet basis (%wb), ultrasonic amplitude (UA), ultrasonic exposure time (UET), methanol (MeOH), water (H_2_O), pomegranate marc peel (PMP), weight for weight (*w*/*w*), solid liquid (SL), half maximal inhibitory concentration (IC_50_), high performance liquid chromatography (HPLC), tannic acid equivalent (TAE), 2,2-diphenyl-1-picrylhydrazy (DPPH), 2,2′-azino-bis (3-ethylbenzthiazoline-6-sulfonic acid) (ABTS), radical scavenging activity (RSA), quercetin equivalent (QE), ferric reducing antioxidant power (FRAP), dry weight (DW), supercritical fluid extraction (SFE), hydrolysable tannins (HT), condensed tannins (CT), pressurised water extraction (PWE), ultra-high performance liquid chromatography tandem mass spectrometry (UHPLC-MS/MS), catechin equivalent (CE), enzyme-assisted supercritical fluid extraction (EASCFE), enzyme-assisted solvent extraction (EASE), microwave-assisted extraction (MAE), ultrasound-assisted extractions in continuous (CUAE), Pulsed ultrasound-assisted extraction (PUAE).

### 4.3. Ultrasound-Assisted Extraction (UAE)

This is one of the useful extraction methods claimed to be more efficient compared to conventional techniques such as hydrodistillation, Soxhlet extraction, and maceration [131,132]. There are a number of studies that have shown the advantages of using ultrasound-assisted extraction with regard to reduced operational time and solvent consumption and increased yield in pomegranate peel [124,126,127,129,131].

Sharayei et al. [127] evaluated and optimised the ultrasound-assisted extraction procedure with water as a solvent to maximise the yield of phenolic compounds and antioxidant activity from pomegranate (cv. Sishe Kape-Ferdos) peel. This investigation determined that the best conditions were an ultrasonic amplitude of 60% and an ultrasonic exposure time lasting 6.2 min. With these optimum conditions, the predicted maximum values of the extraction yield and total phenolic content of the pomegranate peel extract were 13.1% and 42.2 mg GA/g, respectively. Contrary to this, Nag and Sit [128] studied the effects of ultrasound and viscozyme on the extractability of polyphenols from pomegranate (cv. Dalim) peel and to optimise the processing conditions in order to maximise the yield of polyphenols by applying theresponse surface methodology). The optimum conditions for maximum extractability were chosen as an ultrasonication time of 41.45 min and the addition of an enzyme concentration of 1.32 mL/100 mL, with an incubation time of 1.821 h and incubation temperature of 44.85 °C for digestion. The values for the total phenolic content and total flavonoid content under optimised conditions were 19.77 mg gallic acid equivalent (GAE)/g and 17.97 mg QE/g, respectively.

Živković et al. [129] determined the impacts of the extraction temperature, extraction time, solid-to-solvent ratio, and ethanol concentration on the total phenolic content and on contents of individual bioactive compounds (ellagic acid, gallic acid, punicalin, and punicalagin) in pomegranate peel extract. The optimal extraction process conditions were as follows: extraction time of 25 min, ethanol concentration of 59%, solid-to-solvent ratio of 1:44, and extraction temperature of 80 °C. Total phenolic content values in extracts were obtained using the UAE technique, which varied between 81.61 and 190.94 mg gallic acid equivalent (GAE)/g DW (Table 5). Goula et al. [130] tested the extractability of pomegranate peel carotenoids using ultrasound and different vegetable oils as alternative solvents and showed that the highest concentrations obtained were 0.6134 and 0.6715 mg carotenoids/100 g of dry peel using sunflower oil and soy oil, respectively. Furthermore, a single extraction with the green solvents and using ultrasound successfully extracted about 85.7 and 93.8% of the total carotenoids present in the waste material, respectively. Tabaraki et al. [135] optimised the ultrasonic-assisted extraction of natural antioxidants from pomegranate peel using response surface methodology for preparation of food grade extracts. They showed that total phenolic content varied from 5506.42 to 8923.24 mg gallic acid equivalent/100 g of dry weight.

Numerous researchers have used ultrasound-assisted extraction for pomegranate peel (Table 5). Although ultrasound-assisted extraction uses less energy and solvent consumption compared to conventional methods, the process is affected by several variables, which include the frequency, ultrasonic wave distribution, sonication power, and time [128,129]. To evaluate the variables affecting ultrasound-assisted extraction, optimisation approaches must be utilised.

### 4.4. Microwave-Assisted Extraction (MAE)

This relatively novel extraction approach using a microwave applicator as an energy source has received increasing attention due to it requiring less time and less solvent and giving a higher extraction rate, better productivity, and higher quality products at a lower cost [135,136]. Due to these benefits, several studies using microwave extraction technology have been conducted to process pomegranate peel waste (Table 5).

Zheng et al. [138] employed response surface methodology to study the effects of the microwave output power, extraction time, and solid–liquid ratio on total phenolic yield, and determined the optimum parameters to maximise the total phenolic yield of pomegranate peel. The average experimental phenolic yield value under the optimum conditions was found to be 210.36 mg gallic acid equivalent (GAE)/g, similar to the predicted value of 214.46 mg gallic acid equivalent (GAE)/g. It was found that the extract was an effective scavenger in quenching 2,2-diphenyl-1-picrylhydrazyl radicals, with an inhibitory concentration (IC_50_) of 14.53 µg/mL. Huang et al. [139] optimised the microwave-assisted extraction conditions for the extraction of flavonoids from pomegranate peel, with the highest recorded yield for the flavonoids being 4.26%. It was found that the extract was an effective scavenger in quenching 2,2-diphenyl-1-picrylhydrazyl radicals, with an IC_50_ of 14.53µg/mL. Castro-López et al. [140] determined the influence of the extraction method and solid–liquid ratio on the total phenolic content, as well as the antioxidant abilities of four plant materials, namely *Punica granatum* peels, *Juglans regia* shells, *Moringa oleifera*, and *Cassia fistula* leaves. Out of the samples tested, the pomegranate peel extracts using microwave-assisted extraction method had the highest total phenolic content value, which was measured to be 18.92 mg gallic acid equivalent (GAE)/g. In addition, microwave extraction compared to decoction extraction showed important differences, such as in the cinnamic acid value; granatin B were only described in the decoction of pomegranate peels, whereas secoisolariciresinol di-O-glucoside and pedunculagin I were present in the extracted pomegranate peel extracts generated with the microwave-assisted method. It was, thus, apparent that microwave-assisted extraction was more efficient than ultrasound-assisted extraction, decoction, and maceration extraction due to the recovery amounts of phenolic compounds. Furthermore, it is clearly evident that using microwave extraction technology may provide a wider range of phenolic content recovery values than conventional extraction methods. For this reason, microwave extraction technology may serve as an attractive alternative extraction method for commercial use in the recovery of bioactive compounds from pomegranate peel and other fruit waste sources.

### 4.5. Pressurised Liquid Extraction (PLE)

Pressurised liquid extraction (PLE) is also known as accelerated solvent extraction (ASE) or subcritical solvent extraction. PLE is another innovative extraction technique that recovers polyphenols of plants through high temperature and pressure [139,142,144]. Pressurised liquid extraction may serve as a good alternative extraction technique for commercial use in phenolic recovery from pomegranate peel waste. This technique is gaining popularity, as shown in Table 5. For example, Ҫam and Hişil [141] examined the suitability of pressurised water extraction of polyphenols in nine Turkish pomegranate (cv. Izmir 8, Izmir 10, Izmir 16, Izmir 23, Izmir 26, Izmir 1264, Izmir 1479, Izmir 1499, and Izmir 1513) peels, as well as the subsequent use of the extracts as natural antioxidants. They reported that pressurised water extraction compared to conventional methanol extraction took approximately 15 min for total extraction, whereas methanol extraction took 1 h. Moreover, the highest condensed tannin value of 9.5 mg catechin equivalent (CE)/g DW was obtained using pressurised water extraction, while the lowest value of 3.7 mg catechin equivalent (CE)/g DW was obtained using a water extraction method. The highest amount of hydrolysable tannins (262.7 mg tannic acid equivalent (TAE)/g DW) was recorded using the pressurised water extraction technique in comparison to value of 82.6 mg tannic acid equivalent (TAE)/g DW for the water extraction method. Punicalagin was the major phenolic compound recovered from the pomegranate peel using HPLC-DAD at 378 nm, highlighting the effectiveness of pressurised liquid extraction in the recovery of polyphenols in the peel versus conventional solvent extraction using methanol.

Similarly, Sumere et al. [142] evaluated the possibility of combining two extraction techniques (ultrasound-assisted extraction and pressurised liquid extraction, forming ultrasound-assisted pressurised liquid extraction) to improve the efficiency, selectivity, and yield of the extraction of phenolic compounds from pomegranate peel (cv. Wonderful) and the influence of the most important variables in the process. Using a larger peel particle size of 1.05 mm compared to 0.68 mm and using water extraction at a temperature of 70 °C with ultrasound power of 480 W for 3 cycles, the best extraction conditions were, thus, established. Through this protocol, superior levels of phenolic compounds (61.72 mg/g) were recovered from the pomegranate fruit peel. Contrary to this, the use of higher ethanol lowered the phenolic compound extraction yield. Furthermore, it was concluded that it is possible to combine two extraction techniques, such as ultrasound-assisted extraction and pressurised liquid extraction, to enhance the recovery of phenolic compounds from pomegranate peel by manipulating process parameters (Table 5).

Alexandre et al. [143] studied the effects of high-pressure-assisted extraction on antioxidant activity and bioactive compounds of Portugal pomegranate peel, using a Box–Behnken design to evaluate the effects of pressure, extraction time, and ethanol concentration to estimate the optimum extraction conditions by using the response surface methodology. It was evident that high pressures of 300 and 600 MPa led to increases in the total phenolics, with values of 12% and 6% being attained, respectively, which could be attributed to the cell wall breakdown resulting from the high pressure. Furthermore, greater amounts of extract of 6% and 3% were recovered when pressures of 300 and 600 MPa were applied, respectively. The optimum conditions were reported at an extraction time of 30 min, pressure of 492 MPa, and an extraction concentration of 37%. Pressurised liquid extraction is considered a “green technology” that requires small amounts of organic solvents. Operators have the ability to adjust the processing parameters to isolate or select a particular group of compounds. Moreover, it has a faster extraction time compared to other conventional methods [142,144]. This type of technology enables the rapid isolation of valuable compounds from the pomegranate peel and is also environmentally friendly and important for industry, as it saves labour costs and energy. Innovative extraction technologies that can easily be translated to an industrial context from laboratory-based research are, thus, in high demand.

## 5. Conclusions and Future Prospects

This review has highlighted past and present research and development activities in the use of the pomegranate fruit peel, which is a rich source of phytochemicals, organic acids, total sugars, vitamins, and other minerals. Due to the many established health benefits of this type of fruit peel, many opportunities in the cosmeceutical, pharmaceutical, and nutraceutical industries exist due to its value addition potential, which could change the mindset of viewing it as an agricultural waste. The incorporation of bioactive compounds from the pomegranate peel into different products is, thus, foreseen to lead to its profitable commodification. This scenario is highly desirable as an economical and environmentally friendly alternative, solving the current problem of fruit peel waste management.

Despite these benefits, processing of the pomegranate peel using drying and extraction techniques may change the chemical and nutritional properties, and consequently alter the biological strength of the processed pomegranate peel products. To avoid the loss of these phytochemicals and nutritional properties, many researchers have found novel ways to optimise the drying and extraction of these phytochemicals in pomegranate peel. However, these processing techniques are energy-intensive, have long drying and extraction times, and are not all environmentally friendly; many of them are also expensive. This leads to the agro-processing industry being reluctant to adopt them as routine procedures in the manufacture of various pomegranate products. Such waste from industrial food processors can also be changed into more valued resources if the recovery of valuable peel-based chemical components is possible. The advantage here is, thus, two-fold, as not only does the fruit peel waste get converted into value-added products, but also at the same time this practice is more eco-efficient. Valorisation of the pomegranate fruit peel will be of great benefit to the economies of developing countries where pomegranates are currently grown. This will possibly occur through creating novel industries that will bring more job opportunities, supporting the bio-economies of such countries, whilst also reducing the amount of waste discarded into the environment. To understand the impacts of processing factors on the phytochemistry and nutritional properties of pomegranate peel, more studies need to be conducted that directly associate these factors to relevant biological potencies and human health promoting properties. It is clear from this review that at present not all scientific research into fruit peel biology integrates pharmacological assays that show the underlying bioactivities of the peels when they are subjected to different test treatments. In addition, phytochemicals and other nutritional compounds consumed through dietary sources (supplements and fortification purposes) usually undergo considerable processing before being consumed. Research on this topic is also somewhat lacking.

To our knowledge, limited studies have reported the effects of pretreatments of pomegranate peel, even though others have shown the potential to improve the quality and phytochemical recovery of other fruit peels (for details, refer to the work of Boluda-Aguilar et al. [145], Choi et al. [146], Deylami et al. [147], Duarte et al. [148], Nurhuda et al. [149], and Peerajit et al. [150]). Pretreatment of the pomegranate peel has been limited to only enzymatic digestion [120]. With respect to hot water blanching, no studies are presently available in the literature, despite the availability of a number of pretreatment methods, whether it be wet blanching, dry blanching, or mechanical (physical), enzymatic, or chemical pretreatments. Pretreatment processes are typically applied to vegetables and fruits after harvest and prior to further processing, with the aim of enhancing both safety and quality attributes. By doing so, the retention of phytochemicals and nutrients is maximised while reducing the retention of enzymes that cause degradation of fruits and vegetables [151,152,153]. The use of pretreatments may enhance the recovery of phytochemicals and further minimise the drying time, subsequently reducing the loss of heat-sensitive phytochemicals and other nutritional properties in the pomegranate peel during processing.

Future research should focus on exploring pretreatment methods to preserve the phytochemical and nutritional properties of the pomegranate peel in order to produce high-quality value-added products. There is also a need to focus on designing and implementing strategies that will use fruit peel phytochemicals in generating new marketable products. More information will also be required to determine the shelf life and performance of these products during the storage associated with pomegranate fruit peels and peel-derived products. In addition, it will be crucial to ascertain the stability and availability of these functional phytochemicals in processed foods and other useful products, once drying and other similar processes have been employed as part of the production value chain. In our view, such research has a high potential to generate unique and novel applications for the pomegranate peel and “turn waste into gold”.

## Figures and Tables

**Table 1:** Medicinal uses of *P. granatum* L.

Medicinal Uses	Plant Part	Administration	Reference
Vermifuge and anthelmintic	Root and bark	Oral	[46]
Diarrhoea	Peel	Oral	[47]
Blood tonic	Juice	Oral	[48]
Treat vaginal white discharges	Peel	Oral	[49]
Weight loss	Juice	Oral	
Stomach disorders	Peel, bark, and leaves	Oral	[50]
To slow the development of cataracts and anaemia	Seed	Ophthalmic	
Fatigue and hear loss		Oral	
Cure sore throat, dental plaque, dysentery, cholera	Juice	Oral	
Cure haemorrhoid flare ups		Topical	
Stop nosebleeds		Oral and or nasal	
Against diarrhoea, dysentery, and intestinal parasites	Peel and bark	Oral	[51]
Slow the development of cataracts	Juice	Ophthalmic	
Tonic for the heart and throat	Seeds and juice	Oral	
Nose bleeds	Flower juice, peel, and bark	Oral and or nasal	
Gum disease		Oral	
Treat haemorrhoids		Topical	
Wound healing	Leaves and bark	Topical	[52]
Antiparasitic		Oral	
Beneficial in fevers and chronic debility due to malaria	Root	Oral	[53]
Antidiabetic properties	Flowers	Oral	[54]

**Table 2:** Examples of published pharmacological studies on *P*. *granatum* L.

Pharmacological Activity *	Plant Part	Test Method	Details	Reference
**Antibacterial (*Escherichia coli* and *Klebsiella pneumonia*) and Gram-positive bacteria (*Bacillus subtilis* and *Staphylococcus aureus*)**	Peel extracts	Microdilution antibacterial assay	Methanolic peel extracts showed strong broad-spectrum activity against Gram-positive and Gram-negative bacteria, with the minimum inhibitory concentrations (MIC) ranging from 0.2 to 0.78 mg/mL.	[8]
**Antityrosinase properties**	Peel extracts	Spectrophotometric method	Active peel extracts against monophenolase and diphenolase had IC_50_ values of 3.66 and 15.88 µg/mL, respectively.	[8]
**Anti-inflammatory**	Peel extracts	Column chromatography combined with in vitro bioassay-guided fractionation	Pomegranate fractions showed potential nitric oxide (NO) inhibition in lipopolysaccharide (LPS) induced RAW 264.7 macrophage cells and also significantly reduced carrageenan-induced mice paw oedema for 1, 3, 4 and 5 h.	[60]
**Antimicrobial (*S. aureus, P. aeruginosa* and *E. coli*)**	Aril and peel extracts	Agar diffusion	All of the fruit fraction extracts exhibited higher antimicrobial activity on *S. aureus* than *P. aeruginosa*, while *E. coli* was resistant.	[9]
**Antifungal (*Penicillium italicum, Rhizopus stolonifera*, and *Botrytis cinerea*)**	Leaf, peel, and seed extracts	Agar diffusion	The highest inhibitory spore germination (ISG) value was reported at 30.92 ± 2.64% followed by 30.29 ± 2.58% for *Botrytis cinerea* and *Rhizopus stolonifer*, respectively. *Penicillium italicum* showed the lowest inhibition of spore germination at 17.86 ± 2.09%.	[44]
**Antioxidant properties**	Peel powder	2,2′-azino-bis (3-ethylbenzthiazoline-6-sulfonic acid) (ABTS)	Antioxidant levels ranged from 1.8 to 6.8 µmol Trolox equivalent antioxidant capacity (TEAC) per gram bread for fresh bread. Addition of peel powder up to 2.5% *w*/*w* to wheat bread significantly increased its oxidative stability, with no effect on innocuousness as assayed with the brine–shrimp larvae assay.	[61]
**Anticardiovascular properties**	Juice	In vivo experiment, 101 haemodialysis (HD) patients were randomised to receive 100 cc of pomegranate juice containing about 0.7 mM polyphenols three times a week for one year	Consumption of juice yielded significant time response improvements in systolic blood pressure, pulse pressure, triglycerides, and high density lipoprotein (HDL) level. These beneficial outcomes were more pronounced among patients with hypertension, high levels of triglycerides and low levels of HDL.	[43]
**Antihyperglycaemic, antihyperlipidaemic, and antioxidant properties**	Peel extracts	In vivo experiment, 56 Wister albino rats were distributed into 8 groups and compared with standard drugs (glibenclamide and atorvastatin). For antioxidant activity: DPPH (2,2-diphenylpicrylhydrazyl) and ABTS (2,2′-azino-bis (3-ethylbenzthiazoline-6-sulfonic acid)	Peel extracts showed antihyperglycaemic and antihyperlipidaemic activities from a powerful reactive oxygen scavenger through its antioxidant compounds. In addition, the peel extracts enhanced liver and kidney functions when compared to standard drugs in diabetic and hyperlipidaemic rats.	[62]

* Examples are given and this list may not necessarily be exhaustive.

**Table 3:** Phytochemical constituents in different parts of the pomegranate plant (*P. granatum* L.) [45,65].

Plant Part	Constituents
Roots and bark	Ellagitannins, including punicalagin and punicalin, and several piperidine alkaloids.
Leaf	Tannins (which include punicafolin and punicalin), flavone glycosides, luteolin and apigenin, and brevifolin carboxylic acid.
Pericarp (Peel)	Ellagitannins (punicalin and punicalagin), gallic acid and other fatty acids, catechin and epicatechin, quercetin, rutin and other flavonols, flavones, flavonones, procanthocyanidins, and anthocyanidins.
Flower	Gallic acid, triterpenoids, and ursolic acids, including maslinic and asiatic acids.
Seed oil	Punicic acid, hydroxybenzoic acids (gallic and ellagic), other fatty acids, sterols (daucosterol, campesterol, stigmasterol, beta-sitosterol), and γ-tocopherol.
Juice	Procanthocyanidins and anthocyanins, ellagitannins (punicalin and punicalagin), glucose, ascorbic acid, ellagic acid, gallic acid, caffeic acid, catechin and epicatechin, quercetin, rutin, amino acids (methionine, proline, and valine), and many minerals especially irons.

**Table 4:** The effect of drying techniques on the phytochemical and nutritional properties of pomegranate peel waste.

Drying Technique	Cultivar	Country	Phytochemical and Nutritional Properties	Key Findings	Reference
Sun (4 days) and oven drying (overnight at 100 °C)	Baghva, Ruby, Indian white, and Egypt	Sultanate of Oman	Moisture loss (%), pH, and vitamin C	Sun-dried fruit peel had highest water loss, pH, and vitamin C values, with ranges of 64.49–82.52%, 3.5–4.5, and 100–75 mg/100 g dry weight (DW), respectively.	[9]
Freeze-drying (20 °C for 96 h), air drying (48, 30, and 24 h), vacuum drying (60 kPa vacuum at 40, 60, or 90 °C for 24, 13, and 4.5 h), and sun drying for 11 h	Helow	Sultanate of Oman	Moisture loss (%) and total phenolics	Freeze-dried peels showed phenolic contents comparable to those of fresh peel (i.e., 4900 and 4010 mg gallic acid equivalent (GAE)/100 g dry peel solids). Vacuum drying ranged from 1200 to 5330 mg gallic acid equivalent (GAE)/100 g dry peel solids extracted in ethanol at 40 °C and in water at 90 °C, respectively. The highest sun-dried peel value of 4080 mg gallic acid equivalent (GAE)/100 g dry peel solids was achieved in water extract.	[85]
Microwave drying (up to 9 min) air drying (5 days), sun drying (5 days) silica gel drying (up to 7 days) and hot air oven drying (40 °C for 30 h)	Unknown	India	Moisture loss (%)	Highest drying weight for pomegranate peel using microwave dryer at 34.67 g. Lowest moisture loss for pomegranate peel reported using a microwave oven dryer at 65.33%.	[86]
Oven (40 °C for 48 h) and solar drying (50 °C for 2 h)	Unknown	Egypt	Total phenolic content (TPC) and total flavonoid content (TFC)	Total phenolic content (TPC) highest in oven-dried peel with 17.78 mg gallic acid/g in methanol extract. Total flavonoid content (TFC) values statistically similar for both oven- and sun-dried whole peel at 7.98 and 7.96 mg rutin equivalent (RE)/g, respectively.	[18]
Freeze (−88.7 °C for 16 h) and oven drying (40, 50, and 60 °C for 22, 17, and 12 h, respectively)	Wonderful	South Africa	Punicalin, rutin, *p*-coumaric, +catechin,-epicatechin, hesperidin, vitamin C	Higher punicalin and *p*-coumaric values at 60 °C. Freeze-dried (FD) peel had higher rutin, +catechin, -epicatechin, and hesperidin values at 4666.03 mg/kg dry mass (DM), 674.51, 70.56, and 16.45 mg/kg DM, respectively. Vitamin C was reported at ˂30 µg ascorbic acid equivalent (AAE)/g dry mass (DM) using an oven drier at 40 °C.	[34]
Freeze-drying at −80 °C, ambient temperature of ~25 °C, and oven drying at 50 °C	Unknown	USA	Total phenolic content (TPC), α-punicalin, β-punicalin, ellagic acid, α- and β-punicalagin	Higher total phenolic content (TPC), α- punicalin, and β-punicalin values in freeze-dried peel samples of 96 mg/g gallic acid equivalent, 0.8 mg/g, and 1.6 mg/g, respectively. Higher punicalagin (~38.6–50.3 mg/g) and ellagic acid (~2.8–3.2 mg) levels in the peel fractions.	[87]
Freeze-drying (FD) for 48 h; convective drying (CD) at 50, 60, or 70 °C; vacuum–microwave drying (VMD) at 3 different power levels of 240, 360, or 480 W; combined drying (CPD-VMFD) and predrying (CPD) at 60 °C for 90 or 150 min, VMFD with microwave wattage of 360 W.	Mollar de Elche	Spain	Punicalagin isomers (α-PC and β-PC), ellagic acid (EA), and total phenolic content (TPC)	The α-punicalagin and β-punicalagin isomers were highest in fresh peel at 139 and 143 mg/g DW, followed by freeze-dried peel at 113.4 and 98.7 mg/g dry weight (DW), respectively. High ellagic acid (EA) reported in fresh peel at 2.49 mg/g, followed by convective drying (60 °C) and freeze-drying, which were statistically similar at 1.95 mg/g at 1.71 mg/g, respectively. Fresh samples had higher total phenolic content (TPC) values at 125 mg/g gallic acid equivalent, followed by freeze-dried peel at 118 mg/g gallic acid equivalent.	[88]
Single-mode microwave oven dryer, microwave hydro-diffusion (MH), and gravity oven dryer (MHG): both at temperatures ˂ 100 °C for ˂5 min, and traditional oven dryer at 40 °C for 48 h	Acco and Wonderful	Italy	Total phenolic content (TPC), free ellagic acid (EA), and ellagitannins (ETs)	Microwave hydro-diffusion gravity oven dryer was reported with high amounts of total phenolic content (TPC) at 190–230 mg gallic acid equivalent (GAE)/100 mL, ellagitannins at 106–110 µg/mL, and radical scavenging activity (RSA) at 480–560 mg ascorbic acid equivalent antioxidant capacity (AEAC)/100 mL.	[89]

**Abbreviations:** dry weight (DW), total phenolic content (TPC), total flavonoid content (TFC), gallic acid equivalent (GAE), dry mass (DM), ascorbic acid equivalent (AAE), rutin equivalent (RE), Freeze-dried (FD), convective predrying-vacuum-microwave finish drying (CPD-VMFD), vacuum-microwave finish drying (VMFD), ascorbic acid equivalent antioxidant capacity (AEAC), ellagic acid (EA), α-punicalagin (α-PC), β punicalagin (β-PC), microwave hydro-diffusion (MH), Microwave hydro-diffusion gravity oven dryer (MHG), radical scavenging activity (RSA).
